# Risk prediction for emboli and recurrence of primary cardiac myxomas after resection

**DOI:** 10.1186/s13019-016-0420-4

**Published:** 2016-02-02

**Authors:** Zhengjun Wang, Shiqiao Chen, Mei Zhu, Wenlong Zhang, Haizhou Zhang, Hongxin Li, Guidao Yuan, Chengwei Zou

**Affiliations:** Department of Cardiovascular Surgery, Provincial Hospital Affiliated to Shandong University, Jinan, 250021 Shandong Province China; Department of Coronary Care Unit, Provincial Hospital Affiliated to Shandong University, Jinan, 250021 Shandong Province China; Department of Ultrasound, Provincial Hospital Affiliated to Shandong University, Jinan, 250021 Shandong Province China

**Keywords:** Cardiac myxoma, Embolism, Recurrence, Survival

## Abstract

**Background:**

Risk factors for embolism and recurrence of primary cardiac myxoma are not well established. This study aimed to assess the risk factors for embolism and recurrence of cardiac myxoma, as well as the survival of the patients.

**Methods:**

The medical records of 207 consecutive patients treated for primary cardiac myxoma between September 1988 and October 2014 were retrospectively analyzed. All diagnoses were pathologically confirmed. Data were collected to identify the risk factors influencing the prognosis.

**Results:**

Mean age at surgery was 44.2 ± 15.8 years. Operative mortality (within 30 days of the surgery) occurred in seven patients. Mean follow-up was 9.35 ± 6.55 years. Embolism occurred in 32 (15.5 %) patients before surgery. Multivariate analysis indicated that small (≤4.5 cm) myxoma (OR = 5.14; 95 % CI, 2.30–11.94; *P* < 0.0001) and soft, gelatinous myxoma (OR = 5.84; 95 % CI, 1.91–25.61; *P* = 0.001) were independently associated with the occurrence of embolism. Ten patients experienced recurrences. After excluding the patients who died within 30 days of surgery, survival was 92.7 % at 10 years. Age, sex, tumor size, cardiopulmonary bypass duration, aortic cross clamp duration, tumor appearance, and pre-operative embolism were not associated with early mortality. Multivariate analysis showed that multicentric myxomas were independently associated with recurrence (OR = 9.45, 95 % CI, 2.15–41.3, *P* = 0.004).

**Conclusions:**

The surgical resection of primary cardiac myxoma is associated with excellent long-term survival. Tumors ≤4.5 cm and soft tumors were independent risk factors for embolism. Multicentric cardiac myxoma was an independent risk factors for recurrence of myxoma.

## Background

Primary cardiac tumors are rare and have an estimated incidence of 0.5–1.0 cases per 1,000,000 people per year [1]. Primary cardiac myxoma (CM) are the most prevalent type of primary cardiac tumors in adults, representing up to 50–85 % of all benign lesions, while during fetal development, infancy, and childhood, CMs represent only 15 % of cardiac tumors with most of them found in older children [1]. Myxomas most commonly occur between the third and the sixth decade of life and tend to be more common in women [2]. CMs most frequently (60–80 %) affect the left atrium [3, 4].

CMs may cause symptoms either because of intracardiac obstruction, systemic embolism of tumor fragments, or constitutional symptoms by unclear mechanisms [2]. The most feared consequences of cardiac myxomas are preoperative systemic embolism and myxoma recurrence after surgical resection. However, because of the low incidence of primary cardiac myxoma, only few studies have analyzed the risk factors associated with embolism and recurrence of myxomas [5, 6], and there is still a huge need for more clinically relevant data.

Nevertheless, because of their strategic localization and inherent histopathological characteristics, CMs are clinically considered as being malignant entities with very serious consequences in terms of morbidity and mortality. Embolic stroke and heart failure are two significant causes of mortality in patients with CM [1], but the exact factors associated with the occurrence of embolism, early death, or long-term survival are poorly known.

Therefore, the aim of this study was to assess the risk factors for embolism and recurrence of CM, as well as survival of the patients diagnosed with CM between 1988 and 2014 at a single center.

## Methods

### Study design

This was a retrospective study that was carried out at the Department of Cardiovascular Surgery of the Provincial Hospital Affiliated to Shandong University, Jinan, China. This study was approved by the hospital’s Institutional Review Board. Individual consent was waived by the committee because of the retrospective nature of the study.

### Patients

**Fig. 1:**
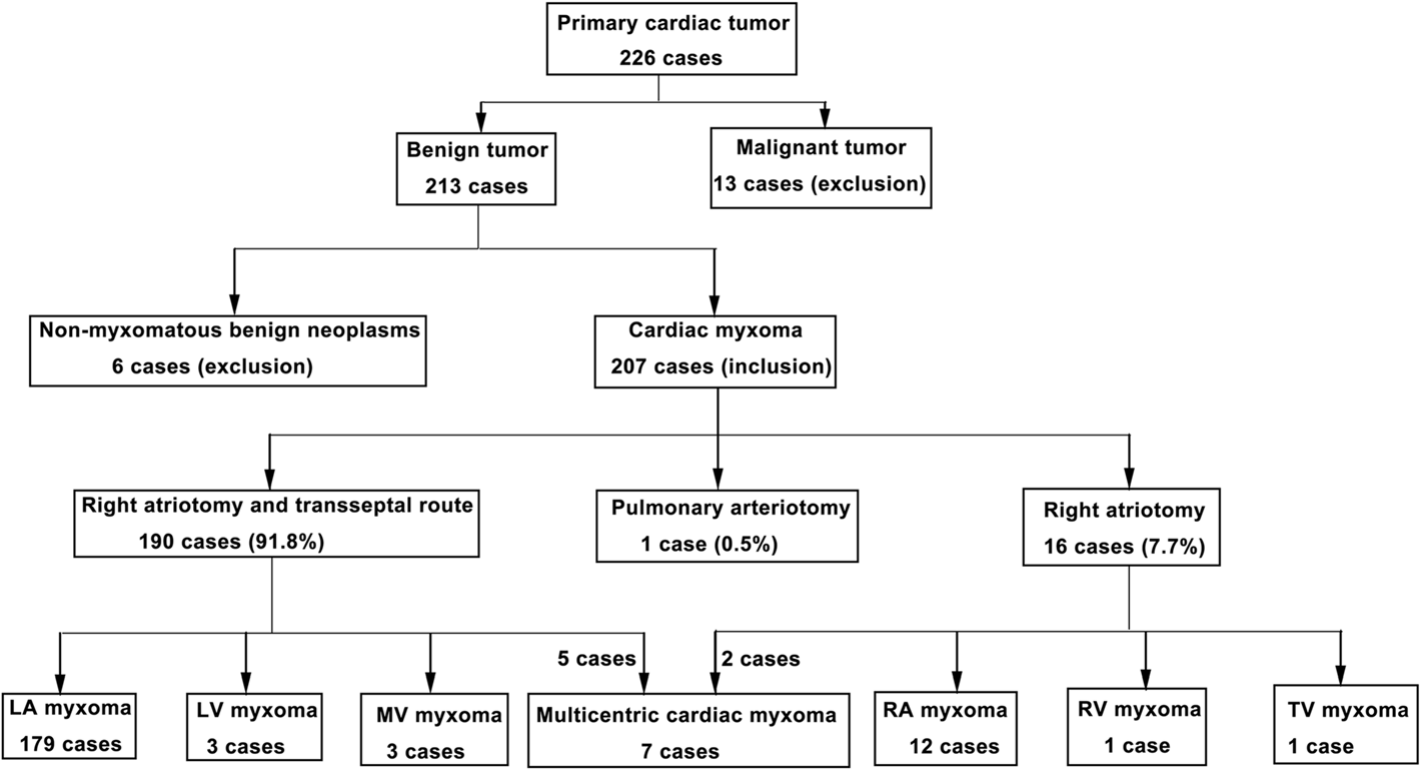
Patient flow chart. The diagram shows patient distribution from enrolment to subgrouping of the included study subjects. Of the 7 cases with multicentric cardiac myxoma, 5 and 2 underwent right atriotomy and transseptal route and right atriotomy, respectively

Patients were included if they had been diagnosed with and treated for CM between September 1988 and October 2014. Patients were excluded if they had non-myxomatous cardiac tumors or if they had a malignant cardiac tumor. Therefore, during this period, 226 patients with cardiac tumor underwent surgery at our hospital including 6 patients with non-myxomatous benign tumors and 13 patients with of primary malignant cardiac tumors, which were excluded. Finally, 207 patients with CM were included (Fig. 1).

### Diagnostic procedures

All patients underwent transthoracic echocardiography and/or transesophageal echocardiography for preoperative diagnosis. Coronary angiography was indicated to investigate coronary artery disease in patients >50 years in order to perform the appropriate coronary treatment at the same time, if needed. Stroke, transient ischemic attack, or other systemic/pulmonary embolic event were diagnosed by magnetic resonance imaging (MRI) or computed tomography (CT) scan. The patients who experienced stroke, transient ischemic attacks, cardiac embolism, or any systemic/pulmonary embolic events were classified as being with pre-operative embolism. CM diagnosis was confirmed by histopathological assessment of surgical specimens.

### Surgery

Surgical resection of intracardiac CM was performed in all patients through median sternotomy accompanied by moderate hypothermia and cardiopulmonary bypass [7]. Myocardial protection was performed by antegrade blood cardioplegia or crystalloid cardioplegia. The surgical approach to CM included right atriotomy and transseptal route in 190 (91.8 %) patients, right atriotomy in 16 (7.7 %) patients, and pulmonary arteriotomy in one (0.5 %) patient.

In 134 (64.7 %) patients, the CM resection was extended to the tumor implantation base, requiring partial resection of the interatrial septum followed by reconstruction using a Dacron or autologous pericardial patch. In two patients with a right atrial CM, the CM was excised on beating heart during cardiopulmonary bypass. The duration of cardiopulmonary bypass was calculated using the data of the patients. The aortic cross-clamp time was estimated according to the overall data of all patients with cardiac arrest.

In order to reduce the risk of local tumor recurrence, resection lines were coagulated with electrocautery. After careful inspection, copious irrigation of atria and ventricles with saline solution was performed to eliminate any tumor fragments.

Concomitant procedures included: mitral valve repair in three patients, ventricular septal defect repair in two patients, atrial septal defect repair in one patient, abdominal artery thrombectomy in one patient, amputation of a lower extremity in one patient, tricuspid valve repair in four patients, and coronary artery bypass grafting in two patients.

During the study period, all surgeries were performed by a total of five surgeons who were experienced in cardiac surgery and had titles of deputy director or above. They had at least 26 years of experience.

### Follow-up

Routine follow-up was performed at 3, 6, and 12 months, and then yearly; after 4 years, follow-up was performed if needed or during a routine check-up. Follow-up data were obtained from subsequent clinical visits, questionnaires mailed to the patients or their family, and/or telephone. Sixteen patients (7.7 %) were lost to follow-up. The last follow-up recorder for this study was in March 2015.

### Data collection

Demographics, disease characteristics, surgery details, and follow-up information were retrieved from the medical charts. Follow-up included patient survival status, cardiac function (New York Heart Association (NYHA) classification), and CM recurrence. The cause of death was recorded.

### Characteristics of the tumors

As per the subsequent receiver operating characteristic (ROC) curve analysis, a tumor ≤4.5 cm in its largest dimension was defined as small CM. Multicentric CM was defined as a CM occurring in multiple heart chambers or originated from different parts of a single cardiac cavity. CMs were divided into solid and encapsulated or soft and gelatinous based on the classification system by Ha et al. [8]. Tumors with smooth regular borders were classified as solid, which was characterized by a round shape and a non-mobile surface. The gelatinous CMs were characterized by a soft and irregular shape and a mobile surface. The gelatinous tumors were more friable, often with a cauliflower appearance necessitating piecemeal removal [9].

### Outcomes

The primary outcomes included death from any cause, heart-related death, CM recurrence, and NYHA status during follow-up. Heart-related death was defined as an occurrence of death due to congestive heart failure or myocardial infarction. CM recurrence was defined as the recurrence of CM after resection.

### Statistical analysis

All statistical analyses were performed with SPSS 17.0 (IBM, Armonk, NY, USA). Continuous variables are presented as mean ± standard deviation and were analyzed with Student t tests. Continuous data are presented as proportions and were analyzed using the chi-square test. The analyses were performed in two steps. First, analyses were performed to assess the factors associated with embolism. Then, analyses were carried out to analyze the factors associated with long-term survival. Univariate analyses were first performed; then, variables with univariate *P*-values <0.05 were included in multivariate logistic regression models. Primary survival analyses were performed with the Kaplan-Meier method and the log-rank test. Cox regression models were used to identify univariate and multivariate predictors of recurrence. Two-sided *P*-values <0.05 were considered significant.

## Results

### Baseline characteristics

Table 1 presents the characteristics of the patients. They were aged 44.2 ± 15.8 years at diagnosis, 65.7 % were female, and 78.5 % were pre-operative NYHA class I/II. The most common symptoms were dyspnea (*n* = 130, 62.8 %), palpitation (*n* = 117, 56.5 %), easy fatigability (*n* = 49, 23.7 %), and fever (*n* = 17, 8.2 %). Thirty-two patients (15.5 %) presented with syncope (*n* = 21) and thromboembolism, including cerebral embolism (*n* = 20, 9.7 %), transient ischemic attack (*n* = 4), renal arterial embolism (*n* = 1), coronary obstruction (*n* = 1), peripheral embolism (*n* = 5), and aorta embolism (*n* = 1). Thirteen patients (6.3 %) were asymptomatic and their CM was an incidental finding. None of the patients had a family history of myxoma nor presented a Carney complex syndrome.

**Table 1:** Characteristics of the patients

Variables	Values
Age, years	44.2 ± 15.8
Sex, n (%)	
Male	71 (34.3)
Female	136 (65.7)
NYHA, n (%)	
III/IV	39 (21.5)
I/II	168 (78.5)
Tumor location	
LA	179 (86.5)
RA	12 (5.8)
LV	3 (1.45)
RV	1 (0.48)
MV	3 (1.45)
TV	1 (0.48)
PV	1 (0.48)
Multicentric	7 (3.38)
CPB time (min)	54.7 ± 25.1
Xclamp time (min)	27.8 ± 18.7
Embolism, n (%)	
Yes	32 (15.5)
No	175 (84.5)
Appearance, n (%)	
Gelatinous	140 (67.6)
Capsulated	67 (32.4)

*LA* left atrium, *RA* right atrium, *LV* left ventricle, *RV* right ventricle, *MV* mitral valve, *TV* tricuspid valve, *PV* pulmonary valve, *IAS* Interatrial septum, Multi = multicentric

Table 2 presents the characteristics of the patients according to CM location. One hundred and seventy-nine (86.5 %) patients had CM in the left atrium, 12 (5.8 %) patients had CM in the right atrium, and seven (3.38 %) had a multicentric disease. One hundred-sixty-seven CM originated from the interatrial septum at the fossa ovalis.

**Table 2:** Characteristics of the cardiac myxomas according to tumor location

Origin	Age (years)	Sex, Female (n)	IAS involvement (n)	Embolism (n)	Appearance Gelatinous (n)	Total, n (%)
LA	46.4	118	158	25	121	179 (86.5 %)
RA	37.1	10	3	2	7	12 (5.8 %)
LV	14.4	1	0	1	2	3 (1.45 %)
RV	18	0	0	0	0	1 (0.48 %)
MV	45.7	2	0	1	2	3 (1.45 %)
TV	7	0	0	0	1	1 (0.48 %)
PV	0.83	0	0	0	1	1 (0.48 %)
Multi	21.6	5	6	3	6	7 (3.38 %)

*LA* left atrium, *RA* right atrium, *LV* left ventricle, *RV* right ventricle, *MV* mitral valve, *TV* tricuspid valve, *PV* pulmonary valve, *IAS* Interatrial septum, *Multi* Multicentric

The tumor size was between 1.0 and 9.4 cm (mean: 5.4 ± 1.8 cm). Solid and encapsulated CMs were observed in 67 (32.4 %) patients and soft and gelatinous CMs were observed in 140 (67.6 %) patients.

### Preoperative embolic events

Table 3 presents the characteristics of the patients according to the presence of pre-operative embolic events. Of the 207 patients with primary CM treated surgically at our institution, 32 (15.5 %) presented in the context of a recent embolic event and met the inclusion criteria: four were with transient ischemic attacks, 28 with stroke and other systemic/pulmonary embolic events before operation. Of the 20 patients with stroke, 12 (60 %) patients suffered from left stroke, 5 (25 %) had right stroke, and 3 (15 %) had multiple strokes. Of the eight patients who experienced a systemic embolic event, five (62.5 %) had an ischemic event in the extremities, one (12.5 %) in the renal artery, one (12.5 %) in the coronary circulation, and one (12.5 %) in the abdominal aorta.

**Table 3:** Characteristics of the patients according to the presence of pre-operative embolism

Variables	Embolism (*n* = 32)	No embolism (*n* = 175)	*P*
Age (years)	39.7 ± 16.6	45.0 ± 15.5	0.079
Gender (male)	12 (37.5 %)	59 (33.7 %)	0.689
Location (left atrium)	26 (81.3 %)	154 (88.0 %)	0.389
Appearance (gelatinous)	29 (90.6 %)	111 (63.4 %)	0.002
Size (cm)	4.45 ± 1.66	5.60 ± 1.72	0.001
Preoperative NYHA Status			0.217
I-II	29 (90.6 %)	139 (79.4 %)	
III-IV	3 (9.4 %)	36 (20.6 %)	

Smaller tumors (4.5 ± 1.7 vs. 5.6 ± 1.7, *P* = 0.001) and a gelatinous tumor appearance (*P* = 0.002) were associated with the presence of pre-operative embolism, while age, gender, tumor location, and pre-operative NYHA status were not (*P* > 0.05).

**Fig. 2:**
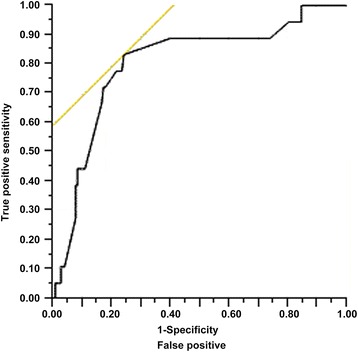
Receiver operating characteristic curve for embolism. The area under the curve was 0.808

In order to evaluate the influence of tumor size on embolism and to determine the best cut-off point for subsequent analyses, a ROC curve analysis (Fig. 2) was carried out. The area under the curve was 0.808 and the optimal cut-off value for tumor size was 4.5 cm according to the Youden Index, resulting in a sensitivity of 83 %, specificity of 75 %, positive predictive value of 38 %, and negative predictive value of 96 %. Univariate analyses showed that tumors of ≤4.5 cm were associated with embolic events compared to tumors >4.5 cm (OR = 4.96; 95 % CI, 2.25–10.97; *P* < 0.0001).

Moreover, a multivariate logistic regression model including patient demographics, tumor location, tumor size, and tumor appearance revealed that small tumor, soft and gelatinous myxomas were the most significant predictors in the occurrence of embolism (Table 4).

**Table 4:** Logistic regression model for the risk of embolism

Variable	Odds ratio	95 % CI	*P*
Tumor size (≤4.5 cm vs. >4.5 cm)	5.138	2.303–11.936	<0.0001
Soft gelatinous myxoma vs. firm capsulated	5.844	1.910–25.613	0.001

### Complications

During the first 3 months after surgery, one patient suffered from excessive postoperative hemorrhage. One patient required tracheostomy for acute respiratory distress syndrome. One patient required permanent pacemaker implantation for grade III atrioventricular block. Five patients showed low cardiac output syndrome. Two patients showed acute renal failure and needed continuous renal replacement therapy. Two patients suffered from postoperative myocardial infarction. Supraventricular tachycardia, pneumothorax, pneumonia, cerebral infarction, and delirium were seen in one patient, respectively.

### Early mortality

Seven patients died before hospital discharge, and the 30-day operative mortality was 3.4 %. The causes of early postoperative mortality were low cardiac output syndrome in three patients, acute renal failure in two patients (one of them died after giving up bedside hemofiltration due to economic hardship, while the other succumbed to co-morbidity of severe pulmonary infection), and postoperative myocardial infarction in two patients.

A multivariate logistic regression logistic regression analysis showed that heart function before surgery (NYHA class III/IV, OR = 6.24, 95 % CI, 1.26–34.5; *P* = 0.026) and age (OR = 1.08, 95 % CI, 1.02–1.17; *P* = 0.009) were associated with early postoperative mortality. Sex, tumor size, cardiopulmonary bypass duration, aortic cross clamp duration, tumor appearance, and pre-operative embolism were not associated with early mortality.

### Recurrences

**Fig. 3:**
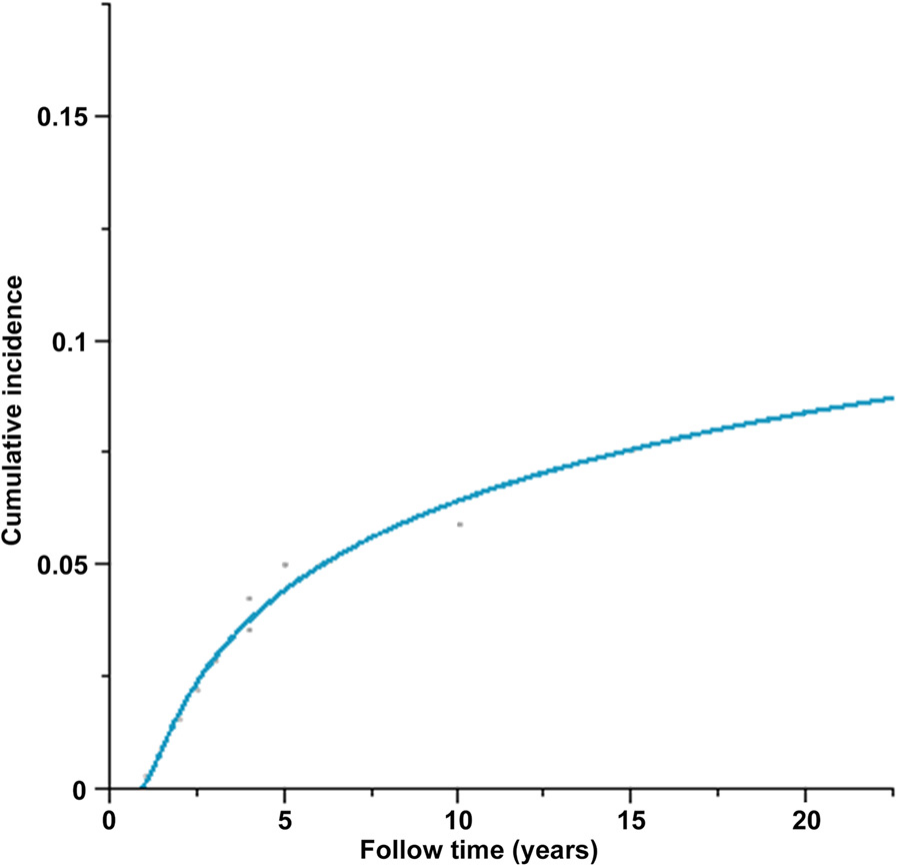
Cumulative incidence of tumor recurrence

Follow-up details were available for 184 (92 %) patients who survived the first month after surgery. Mean follow-up time was 9.35 ± 6.55 years. During follow-up, ten patients had a recurrence diagnosed 1 to 15 years (mean of 4.8 ± 4.4 years) after their primary operation (Table 5). Among these 10 patients, seven had a recurrence in the first 4 years after surgery. The rates of freedom from recurrence at 5, 10, 15, and 20 years were 95.5 %, 93.5 %, 92.4 %, and 91.6 % (Fig. 3). Interestingly, 3 recurrent cases were observed among the 7 cases of multicentric cardiac myxoma, indicating a recurrence rate of 42.3 %. Multivariate analysis showed that multicentric myxomas were independently associated with recurrence (OR = 9.45, 95 % CI 2.15–41.3, *P* = 0.004)

**Table 5:** Tumor recurrence characteristics in the 10 patients with recurrence

No.	Sex	Age at onset	Primary tumor site	Recurrence time (years)	Recurrent site
1	F	32	LA	4	LA
2	M	25	LA	10	LA
3	F	33	LA	1	LA
4	F	21	LA	1	LA
5	F	10	LA,RA	4	LA,LV,RV
6	M	54	LA	15	LA
7	F	11	LA	3	LA
8	F	13	LA,RA	2	LA
9	F	37	LA	7	RV
10	F	22	LA,LV	1.5	RA

### Long-term survival

**Fig. 4:**
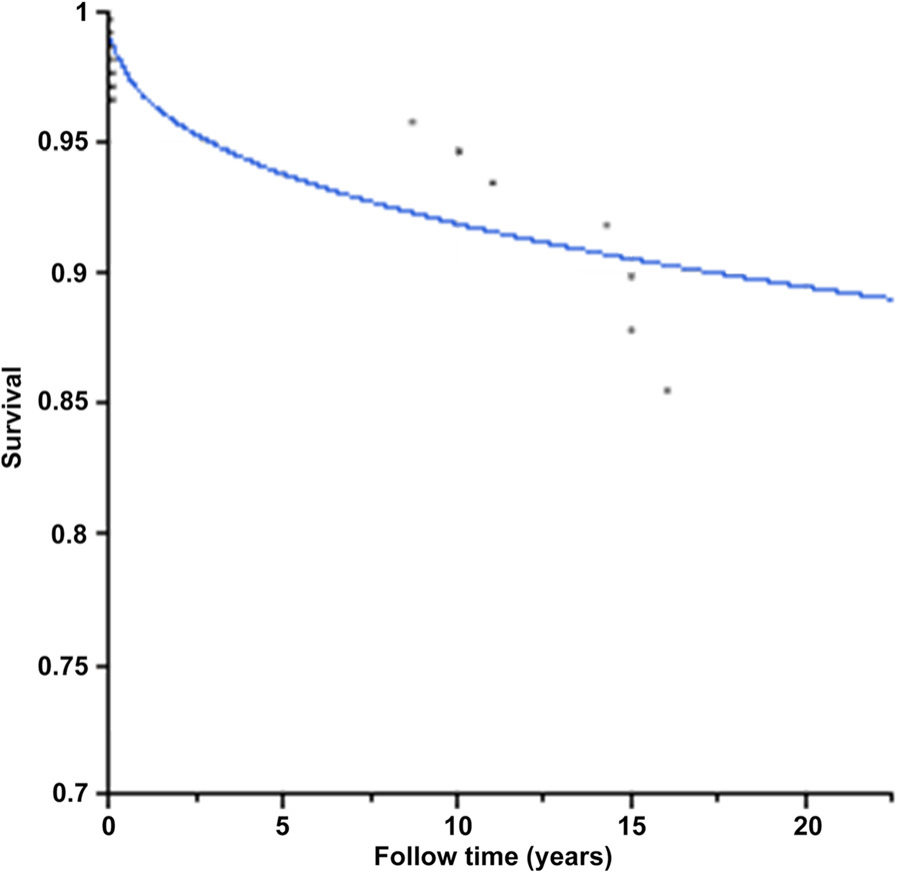
Kaplan-Meier estimate of the survival of patients after surgical resection of their cardiac myxoma

There were seven deaths (3.5 %) during follow-up: three patients died from non-cardiac malignant tumors, one from upper gastrointestinal bleeding, one from a traffic accident, and two from heart-related causes (one from myocardial infarction and another from heart failure). No death could be directly associated with CM. The survival was 94.3 %, 92.7 %, and 91.5 % at 5, 10, and 15 years, respectively (Fig. 4).

Multivariate analysis demonstrated that older age at operation was the only significant predictor of mortality (HR = 1.09, 95 % CI: 1.04–1.15, *P* = 0.001). There was no significant difference in survival when patients were stratified by sex (female, HR = 1.67; 95 % CI: 0.495–5.523, *P* = 0.441), tumor size (HR = 1.00; 95 % CI: 0.73–1.38, *P* = 0.645), and tumor appearance (HR = 2.22, 95 % CI: 0.69–7.17, *P* = 0.158).

## Discussion

The aim of this study was to assess the risk factors for embolism and recurrence of CM, as well as survival of the patients. Results showed that embolism occurred in 32 (15.5 %) patients before surgery. Multivariate analysis indicated that Small (≤4.5 cm) CM and soft, gelatinous CM were independently associated with the occurrence of embolism. After excluding the patients who died within 30 days of surgery, survival was 92.7 % at 10 years. Multivariate analysis showed that multicentric myxomas were independently associated with recurrence.

Previous reports have estimated that the risk of embolism associated with CM ranged from 30 to 40 %, and that >50 % of embolic events affected the central nervous system and retinal arteries [3]. Another study showed that about 13 % of patients experienced CM recurrence, mostly in the first 4 years [10]. This study showed that the overall rates of tumor embolism and recurrence were 15.5 and 5 %, respectively.

CMs represent the most common type of primary cardiac tumors. Due to their strategic localization and inherent histopathological characteristics, they are clinically considered as malignant entities with very serious consequences in terms of morbidity and mortality. The cells giving rise to CM are considered to be multipotent mesenchymal cells, which persist as embryonal residues during septation of the heart and differentiate into endothelial cells, smooth-muscle cells, angioblasts, fibroblasts, cartilage cells, and myoblasts [3]. They usually are attached to the interatrial septum at the border of the fossa ovalis area, and 75–80 % of CMs occur in the left atrium [3, 11], which is consistent with this study.

Embolism is a well-known complication of CM. Embolic manifestations have been observed in 30–50 % of patients with CM. They are caused by tissue fragmentation, detachment of tumor, and/or dissemination of overlaying thrombi or foci from the tumor surface. Therefore, the American Heart Association guidelines propose that during evaluation of an embolic event, intracardiac masses should be suspected and appropriately pursued with echocardiography based on a suspicious clinical presentation [12]. Due to the prevailing left-sided location of CMs and high pressure within the left ventricle during systole, systemic embolisms (cerebral and peripheral) are most frequently encountered in cerebral and retinal arteries, followed by arteries of the lower extremities, visceral, renal and coronary arteries, and sometimes even in the abdominal aorta [13]. He et al. think that tumor location, macroscopic appearance, mean platelet volume, and high platelet count are strong risk factors for embolic events in patients with cardiac myxomas [14]. Elbardissi et al. [5] have suggested that patients with small tumors, minimal symptoms, and no evidence of mitral regurgitation have a high risk of embolism. This study confirmed these observations and the results are consistent with reported values [15, 16].

Although coronary embolism is extremely rare, it should be considered even in patients with no cardiac risk factors [17, 18]. One of the patients of this present study had a left atrial myxoma and had no cardiac risk factors, but she suffered from acute angina and presented with inferior myocardial infarction secondary to tumor embolism.

Since Gerbode et al. [19] first described CM recurrence, there have been a number of reports describing patients with multiple recurrences occurring predominately in patients with CM. The cumulative incidence of CM recurrence is about 1 to 3 % in sporadic forms, 12 % in familial forms, and 22 % in complex forms [20]. This study reported an overall rate of myxoma recurrence of 5 %. About 70 % of the recurrences happened in the first 4 years after operation, but two recurrences happened at 10 and 15 years after the first resection. The relationship between local intracardiac recurrence and the adequacy of surgical resection is quite controversial. It appears that recurrent tumors often do not recur at the site of the original lesion even in patients in which a complete excision could not be done. Furthermore, a large series of postsurgical follow-up reported no recurrent tumors [21]. Shinfeld et al. [22] and McCarthy et al. [23] have suggested that an inadequate resection is the most common cause for recurrent CM arising from the atrial septum. Therefore, resection of full thickness wall with the tumor should be performed with good safety margins whenever possible. Jones et al. suggested the following principles to minimize recurrences [24]: 1) minimal manipulation of the tumor; 2) adequate exposure for complete resection of the tumor; and 3) inspection of all four heart chambers. Gosev et al. [1] indicated that the biological characteristics of CM might be the most reliable factors for recurrence prediction, rather than their overall histopathological presentation. Shah thinks that younger age at surgery, smaller tumor dimension and tumor localized to the ventricles were predictors of recurrence [25].

In this study, all patients had a complete excision, and only the growth characteristics (multifocal vs. single mass) were associated with recurrence. Moreover, the results showed that there were no age differences in patients with or without embolism, which is inconsistent with a previous study [5]. Unfortunately, no DNA testing was performed in this study.

Previous studies have reported a short-term mortality rate of 3.2 % and overall 10-year survival of 96.8–98 % [9, 26], which is consistent with this study. In this study, early death occurred in 3.4 % of patients and only 28.6 % of late deaths were attributed to heart diseases.

This study is not without limitations. Since this study was retrospective, the analysis was subjected to multiple potential biases, and the patients did not have scheduled follow-up echocardiograms. In addition, not all patients were follow-up for 10 years, and we had to rely on the actuarial 10-year survival. Furthermore, the time span of the study period was large, and we cannot exclude a bias due to evolving surgical and patient care technologies. In spite of these limitations, the present retrospective analysis could provide some valuable information on CM.

## Conclusions

In conclusion, embolism and recurrence are the two features that could affect the outcomes of patients with CM. Due to the risk of embolism, surgery should be performed as soon as possible after diagnosis, especially in patients with tumor ≤ 4.5 cm. Multicentric growth is a risk factor for recurrence, which mostly occur in the first 4 years after resection.

### Ethics approval and consent to participate

This study was approved by the Provincial Hospital Affiliated to Shandong University’s Institutional Review Board. Individual consent was waived by the committee because of the retrospective nature of the study.

### Availability of data and materials

The data set supporting the results of this article are included within the article and its additional file.

## Abbreviations

CM: Cardiac myxoma
CT: Computed tomography
MRI: Magnetic resonance imaging
NYHA: New York Heart Association
ROC: Receiver operating characteristic
